# Supplementary material for: Are carbon emissions trading and green financial instruments synergistic? -Comprehensive quantitative research based on content analysis
Source: PLoS One. 2024 Mar 7;19(3):e0298601. doi: 10.1371/journal.pone.0298601 (PMC10919601; doi:10.1371/journal.pone.0298601)
Supplement: S1 Data — (ZIP) [file pone.0298601.s001.zip › Data/地级市Z_F工作报告.zip!/╣π╢1⁄2/╘╞╕í╩╨/└┤╫╘╣1⁄2╓┌║┼í╛┬φ┐╦╩2╛▌═°í┐_2009.docx]

各位代表：

我代表市人民政府向大会作政府工作报告，请予审议，并请政协各位委员和其他列席人员提出意见。

2008年工作回顾

2008年是我市经济社会发展经受严峻挑战并取得显著成绩的一年，我们接连经历了各种自然灾害和国际金融危机的重大考验。市政府在省委、省政府和市委的正确领导下，在市人大及其常委会和市政协的监督、支持下，坚持以科学发展观为指导，团结带领全市人民解放思想、顽强拼搏、抢抓机遇、开拓创新，沉着应对种种困难和挑战，全市经济社会继续保持平稳较快发展，基本完成了市四届人大三次会议提出的各项目标任务。

全市完成生产总值319.86亿元，比2007年（下同）增长10.2%；人均生产总值13439元，增长9.1%；单位GDP能耗下降3.5%；社会消费品零售总额100.6亿元，增长20.5%；全社会固定资产投资总额164.1亿元，增长17.6%；外贸进出口总额11.6亿美元，增长10.2%，其中出口总额7.1亿美元，增长4.9%；地方财政一般预算收入16.63亿元，增长19.1%；城镇居民人均可支配收入12339元，增长5.8 %；农村居民人均纯收入5491元，增长13.8%（扣除物价上涨因素实际增长7.6%）；居民消费价格总水平上升5.9%；人口自然增长率5.32‰；城镇登记失业率2.6%。全市呈现经济增长较快、环境日益优化、民生切实改善、社会事业健康发展、社会大局和谐稳定的良好局面，具体体现在：

——新型工业化发展迈出新步伐。以落实“双转移”战略为抓手，大力推进与佛山市合作共建佛山（云浮）产业转移工业园的产业发展规划、资金筹措、配套设施建设以及项目入园等工作，目前正全力申报省示范性产业转移园；新成产业转移工业园、罗定双东环保工业园、省市共建硫化工基地和其他园区规划建设加快推进。委托国家开发银行广东省分行编制全市产业中长期发展规划，确定了打造石材、不锈钢制品、水泥、硫化工、电力五大年产值超百亿元特色产业集群发展目标。全市35个重点项目累计完成投资40.5亿元，增长65.7%。云浮发电厂C厂、中材(罗定)水泥项目相继动工；华润西江发电厂已列入国家有关部门的审批程序；云浮风电场、云浮核电站和粤泷电厂二期等项目论证申报工作取得积极进展。新增国家驰名商标1个、省名牌产品4个、省著名商标3个。新增私营企业522户。全市规模以上工业实现总产值314.77亿元，增长20.3%；增加值94.53亿元，增长11.9%。

——农业和农村经济呈现新气象。成功举办“温氏模式与农业产业化发展高峰论坛”，进一步推广和发展温氏模式。全市产业化经营组织发展到195个，增加30个；实现销售收入195亿元，增长30%，带动合作农户户均纯增收5400元。温氏集团被列入省“千百亿”名牌培育工程企业，其优良种猪体细胞克隆技术研究应用课题取得新突破；“郁南粤西地区现代蚕桑技术推广与产业化项目”和“新兴华南丘陵山区循环农业技术产业化示范项目”被列为国家级富民强县项目。完成造林作业面积7.43万亩，新兴创建林业生态县通过省验收。完成省人大小型水库除险加固和小水电议案建设任务。开发补充耕地9.57万亩，我市获得省年度土地利用计划执行考核一等奖。粮食播种面积185万亩，总产量68.8万吨，均超额完成省定任务，罗定被评为全国粮食生产先进县(市)。肉类和水产品总产量分别增长12.1%和8%，水果、花卉、蔬菜等主要经济作物获得丰收。外出务工劳动力达52万人，实现劳务收入31亿元，增长22.3%。全市实现农业总产值142.43亿元，增长7.2%。

——第三产业实现新发展。加大服务业招商和项目建设力度，着力推进商贸、旅游、房地产和物流等行业发展。新增“万村千乡”市场工程农家店70家、配送中心2家。成功举办第二届旅游文化美食节和云浮（佛山）旅游推介会等活动，全年接待游客435.9万人次、实现旅游收入34.45亿元,分别增长11%和11.4%。房地产开发投资总额增长25.9%。公路、水路货物周转量和港口货物吞吐量分别增长40.9％、54％和34.4 %。保险机构保费收入增长39.3%。通信、中介等服务业发展步伐加快。全市第三产业实现增加值90.8亿元，增长12.6%。

——交通建设实现新突破。积极抢抓国家和省加大交通基础设施建设力度的有利时机，大力推进交通项目建设和申报立项工作。广梧高速公路河口至平台段累计完成总投资的56.4%，全省最长的高速公路隧道石牙山隧道已贯通；云浮至岑溪高速公路粤境段立项所需前期工作基本完成，将于今年动工建设；江门至罗定、罗定至阳春高速公路经过努力争取，省已同意提前启动建设并委托地方开展前期工作。云浮新港六都四围塘码头累计完成总投资的74.3%，将于今年6月建成，9月投入使用。南宁至广州高速铁路云浮段正全面开展征地拆迁工作，控制性工程郁南花培岭隧道已动工建设。西江航道整治工程已经动工。国道324线大修罗梅段已动工，云城、云安段立项报批工作基本完成。河杨公路扩建与沿江公路建设方案基本确定。此外，对一批省道、县道和农村公路进行建设改造。全市交通基础设施建设完成投资27.3亿元，增长95%。

——宜居城乡建设取得新进展。完成城市总体规划修编纲要，确定了中心城区“北拓东进”发展战略，为做大做优城市奠定了良好基础。市区一批重点工程相继建成使用，石材企业搬迁工程正在抓紧实施；城乡水利防灾减灾工程建设步伐加快，南山河城区防洪工程初步设计获省水利厅批准。完成2个省级试点镇控制性详细规划和79条省级村庄规划试点，建成一批镇村基础设施项目，创建省级生态示范镇2个，新建生态文明村602条，改善了镇村风貌。

——招商引资和外经贸工作创出新业绩。制订完善招商引资优惠政策，并向佛山、广州、深圳、东莞、中山、珠海等市派驻由市领导担任组长的招商小组。成功举办不锈钢制品博览会、云浮(佛山)产业转移推介会和第五届国际石材科技展览会，积极参加国家和省组织的各类经贸招商活动。全年各种经贸活动共签订投资额500万元以上项目93个，投资总额96.7亿元；贸易项目105个，贸易总额31亿元。积极应对金融危机，引导外向型企业调整经营战略，着力培育新的出口企业，鼓励和支持民营企业出口。新批自营进出口权民营企业25家，新增出口额500万美元以上企业3家。

——财税金融工作取得新成效。加强税源财源培植和征管，完成国税收入15.35亿元、地税收入12.96亿元,分别增长11%和15.8%；财政总收入41.87亿元，增长10.7%。坚持公共财政理念，重点保障社会事业、民生工程、重点项目支出，全年财政总支出41.25亿元，增长11.4%，其中一般预算支出39.97亿元，增长10.7%。积极整合土地、矿产及优质项目资源，主动加强与国家开发银行、农业发展银行等金融机构的联系，大力引导市内金融机构支持经济和社会事业发展。全市金融机构本外币存款余额335.4亿元, 贷款余额138.56亿元，分别比年初增长18.9%和27.8%，增幅居全省第6位和第1位。

——各项民生工作有新提高。新增就业人数3.1万人，城镇下岗失业人员再就业0.83万人。组织农村劳动力技能培训1.85万人，新增转移农村劳动力4.69万人。为劳动者追回拖欠工资1753万元。征缴社保费6.5 亿元，增长28%。全市城乡困难居民实现应保尽保，受惠群众5.7万人。动工建设农村饮水安全工程16宗，受惠群众14.45万人；完成13条移民村改造建设任务。新建廉租房3182平方米，全市394户城镇低收入家庭实现住有所居。帮助3.5万名农村贫困家庭子女、非户籍子女解决入学困难。为635名困难群众提供法律援助。新型农村合作医疗参合率99.46%，我市被授予全省农村合作医疗工作先进集体。城镇居民医疗保险参保人数35万人，完成省定年度参保任务。完成省下达关停小火电机组任务，建成云浮发电厂和粤泷电厂脱硫工程，淘汰一批落后水泥产能。提前实现县县建成污水处理厂目标，全市空气环境质量保持在国家二级标准以上，我市在年度珠江综合整治考核中位列全省第1名。整顿和规范市场经济秩序，落实企业治乱减负工作，严厉打击传销和商业欺诈等行为。加强产品质量和食品、药品安全整治监管，严格落实安全生产责任制，重特大安全事故得到有效遏制。加强地质灾害隐患治理和巡查，全年未发生因地质灾害造成的人员伤亡。加强三防工作，全市水利工程实现安全度汛。全面完成公安“三基”工程和司法所办公用房建设，有效遏制“两抢一盗”等恶性犯罪，刑事发案率下降25.6个百分点。进一步加强基层民主自治，全市有15个社区被授予省“六好”平安和谐社区。加强人民调解和社会矛盾排查调处，落实信访条例和工作责任制，群众反映的突出问题得到有效化解。

——各项社会事业取得新进步。与国内高校和科研院所共建产学研创新联盟5个、示范基地3个；组建省级工程技术研发中心2个、省级技术创新专业镇3个；组织实施省级以上科技项目110项，获省级专项资金额列全省第7位。义务教育各项指标均达到省要求；高中阶段教育毛入学率提高10个百分点，其中职业技术教育在校学生增加24.7%；高考上线率和录取率分别达79.1%和69.7%；落实了市教育园区选址，启动了市特殊学校建设工作。创新文化机制，规范文化市场管理，进一步完善了市博物馆、图书馆等公共文化设施；举办了2008广东禅宗六祖文化节和广东省石艺创意大赛等文化活动；云城区和郁南连滩镇分别被评为中国民间文化艺术之乡，云浮国际石材城被评为广东省文化创意产业园。积极开展迎奥运全民健身活动，参加省级以上比赛获得奖项折合省十三运金牌6枚。完成村卫生站规范化建设150间；完善医疗救治、疾病预防、卫生监督、社区卫生四大体系建设，全市无发生人禽流感、手足口病等重大传染病疫情暴发流行；初步理顺了全市血站管理体制；创建省卫生城市灭鼠灭蝇工作通过省考核验收。人口出生率控制在10.77‰以下；人口出生性别比为107.31，下降0.08。成功创建省双拥模范城市。

——民主法制和政府自身建设上新水平。认真落实市人大及其常委会各项决议、决定并向其报告工作，加强与人民政协的联系并向其通报情况，办理人大建议96件、政协提案192件。密切与工会、共青团、妇联等人民团体的联系，广泛听取各民主党派、工商联、无党派人士的意见建议。认真实施“五五”普法教育，深入贯彻落实《公务员法》、《行政许可法》、《全面推进依法行政实施纲要》和《行政首长问责暂行办法》等法律法规。建成市、县、镇三级政府行政服务中心，开通县级行政电子监察系统和镇村电子监察系统。建立健全土地管理、应急管理等机制，认真组织查处整治违法违规用地，进一步规范预算执行审计、财政资金绩效评估、政府采购与建设工程招投标等制度，严格执行廉政建设各项规定。加强政风行风、行政效能和公务员队伍建设，机关作风进一步改善，办事效率进一步提高。

此外，物价、统计、人事人才、编制、经济发展研究、知识产权、民族宗教、档案、外事侨务、台务、港澳事务、人民防空、地方志、消防、保密、气象、扶贫、工会、共青团、妇联、侨联、文联、残联、社科联、工商联、红十字会、贸促会、广播电视、新闻出版以及驻市外机构等工作也取得新成绩。

各位代表！过去一年政府工作取得的成绩，来之不易。这是在省委、省政府和市委正确领导下，全市上下抢抓机遇、开拓进取、共克时艰、奋力拼搏的结果。在此，我代表市人民政府，向全市人民、驻云浮部队官兵和关心支持云浮发展的港澳台同胞、海外侨胞及国际友人表示衷心的感谢并致以崇高的敬意！

同时，我们也清醒地看到，我市经济社会发展仍然存在不少困难与薄弱环节，主要是：世界金融危机给我市经济增长的负面影响仍将持续；交通、城市、园区等基础设施滞后；重大项目特别是工业项目较少，产业结构欠合理；经济总量小，财政收支矛盾突出；城乡居民持续增收难度大，部分低收入群众生活仍较困难；政府自身建设有待加强等。对于这些困难与问题，我们将高度重视，采取有效措施认真解决。

2009年工作安排

各位代表!今年是建国60周年、建市15周年，也是我市建设“广东富庶文明大西关”的开局之年，做好今年工作具有特别重要的意义。当前国际金融危机不仅本身尚未见底，而且对实体经济的影响正进一步加深，其严重后果还会进一步显现。与全国全省各地一样，我市经济受金融危机的影响越来越明显，特别是外向型企业和利用外资工作面临的环境将更加严峻。但在挑战与困难中，也蕴藏着机遇和希望。从国内、省内的大环境看，中央和省都明确要求今年要把保持经济平稳较快发展作为首要任务，这都将为我们有效应对国际金融危机对经济增长所带来的负面影响提供有力的政策支持。从我市自身情况看，经济发展水平和对外依存度都相对较低，受国际金融危机影响也相对较小,经济平稳较快增长的基本趋势没有改变；又是省确定的重点发展地区之一，正处于加快基础设施建设的重要时期，国家和省加大投资、扩大内需政策的实施，必将为我市加快推进基础设施建设，加快改善投资发展环境创造前所未有的机遇。从企业发展的情况看，危机带来的优胜劣汰、人才资金技术流动、出口市场趋紧等，也将推动企业增强危机意识、品牌意识、创新意识和变革意识，促进企业转变生产方式和经营方向，加快生产资源整合升级。只要我们认清形势、坚定信心，充分发挥自身优势，着力解决突出问题，化危为机、克难奋进，就一定能够推动全市经济社会又好又快发展，为迎接新一轮的发展高潮打牢基础。

2009年政府工作的总体要求是：高举中国特色社会主义伟大旗帜，深入贯彻落实科学发展观和党的十七大、十七届三中全会、中央经济工作会议、省委十届四次全会、省十一届人大二次会议精神，按照汪洋同志视察云浮的指示要求和市委四届五次全会的部署，围绕“两区四城一纽带”战略目标和“三年打基础、五年上台阶、十年大发展”战略步骤，全面实施《云浮市改革发展规划纲要》，以解放思想为引领，以改革开放为动力，以加快发展为首要任务，全力加快交通、城市建设和工业、农业、第三产业发展，全面加强统筹发展与制度创新、招商引资与外经贸、财税金融、和谐社会和政府自身建设，为打造“广东富庶文明大西关”开好局、起好步。

2009年经济社会发展主要预期目标是：生产总值增长10.2%，人均生产总值增长9%，单位生产总值能耗下降3.5%，二氧化硫排放量削减至4万吨，化学需氧量排放量控制在2.2万吨以内，全社会固定资产投资增长30%，进出口总额增长10%，实际利用外资增长10%，社会消费品零售总额增长18%，居民消费价格总水平涨幅控制在4%以内，地方财政一般预算收入增长12%，城镇登记失业率控制在3.5%以内，人口自然增长率控制在7.2‰以内，城镇居民人均可支配收入增长10%，农村居民人均纯收入增长7%。

为实现上述目标,必须做好以下十个方面的工作：

一、加快新型工业化进程，提升产业集聚力

着力建设工业园区。继续加强与佛山市合作，抓紧完善佛山（云浮）产业转移工业园总体规划、控制性详细规划、产业规划、土地利用规划、环评报告和相关材料，确保今年成功列入省第三批示范性产业转移园；加快完善园区各项基础设施，加强项目入园工作。继续抓好新兴新成产业转移工业园、罗定双东环保工业园和其他工业园区规划建设和招商引资工作，使园区真正成为承接产业转移的主要载体。

着力发展产业集群。提升石材、不锈钢制品产业，发展水泥、硫化工和电力产业，打造五大年产值超百亿元产业集群。今年重点在石材行业培育一批龙头企业，推进石材博览中心和国家石材检测中心建设，打造行业区域品牌；在不锈钢制品行业提升产品结构和档次，拓展国内外市场，培育上市龙头企业；在水泥行业加快资源整合和淘汰落后产能，加快中材罗定、中材亨达、广信青洲水泥项目和罗定—云安水泥生产基地建设；在硫化工行业强化循环经济理念，着力拉长产业链条，加快省市共建硫化工基地建设；在电力行业抓好云浮电厂C厂建设和云浮风电场、华润西江电厂、云浮核电站、粤泷电厂二期等项目申报工作及郁南抽水蓄能电站前期研究工作，年内争取云浮风电场动工建设,争取华润西江电厂一期项目获得国家批准开展前期工作。同时，在电子、电池行业积极争创省级产业集群升级示范区，依托佛山（云浮）产业转移工业园加快发展机械装备产业，依托温氏生物科技园加快发展生物制药产业，积极开展适合云浮的产业发展可行性研究，培育新的产业集群。

着力扶持中小企业。认真贯彻落实省政府《关于促进中小企业平稳健康发展的意见》，实行领导挂点联系企业制度和定点企业跟踪制度，切实提高经济运行监测预报水平。加强政银企合作、创业辅导、检验检测和信息物流等平台建设，创新中小企业融资渠道，设立中小企业发展专项资金，切实减轻企业负担，改善中小企业和民营经济发展环境。重点扶持一批成长性好、创新能力强、有自主品牌的企业实现转型升级，年内培育申报国家级企业技术中心1家、省级2家，更好地发挥中小企业和民营经济的生力军作用。

二、加快交通基础设施建设，提升区位竞争力

着重推进“四三二一”工程建设。按照动态管理、梯次推进、滚动发展和“在建一批、报审一批、论证一批、储备一批”的工作思路，精心谋划和加快推进以“四三二一”工程为重点的交通基础设施建设。加快在建工程进度，确保云浮新港六都四围塘码头在今年上半年建成，9月底前投入使用；争取广梧高速公路河口至平台段在今年底基本建成，明年初通车；加快南宁至广州高速铁路云浮段建设，抓紧全线征地拆迁工作，年内全面动工建设；加快西江航道整治；努力争取罗定至岑溪铁路纳入国家铁路网络建设。年内动工建设云浮至岑溪高速公路、都骑通用码头；完成江门至罗定、罗定至阳春高速公路、都杨码头立项等前期工作，争取明年动工建设。

着重完善“大西关”交通网络。在重点推进“四三二一”工程建设的同时，加快与之相配套的国道、省道和县乡公路建设。年内完成国道324线罗定段路面大修,动工大修云城、云安段，动工改建安塘至河口段；完成河杨公路河口至佛山(云浮)产业转移工业园（都杨）段扩建，并动工扩建至沿江公路段；动工建设沿江公路六都至都杨段；完成300公里县乡公路建设改造，改善城乡交通条件；完成云浮汽车客运站等5个客运站建设改造，加快发展城乡公共交通事业。争取用五年时间构建好“大西关”主要交通网络。

三、加快生态宜居城乡建设，提升城市辐射力

做大做精中心城区。按照“北拓东进”战略年内完成城市总体规划修编，开展城区功能布局与专项规划。拉开城市建设基本框架，加快推进城市道路交通、治污保洁、供水供气、文化休闲、教育医疗“十项工程”，年内完成创建省级卫生城市、南山河城防首期和星岩路、解放路与浩林西路改造等6项工程，动工建设公共活动场所、市教育园区、市人民医院新院等4项工程，加快完善市政基础设施。同时，继续抓好中心城区石材企业搬迁、综合市场改造和城市建设用地遗留问题调处、土地储备开发等工作。加强城市管理，大力治理占道经营、乱张贴、乱停放以及其他不文明行为，美化、净化城市环境。着力把云浮打造成颇具特色的生态精品宜居城市。

做优做美城乡环境。坚持生态优先、环境优先的原则，开展创建生态宜居城乡活动，努力争取列为全省生态示范市试点。严格执行节能问责制和主要污染物排放总量控制制度，创建循环经济示范区；严格执行土地管理问责制，强化节约集约用地。着手推进云城与云安同城化及资源共享，加强县城与中心镇规划建设和管理，完善城乡供水、供电、通信、污水与垃圾处理等基础设施，引导农村人口向城镇集聚，加快城乡一体化进程。以“百镇万村”村镇建设规划活动为切入点，以创建林业生态市和建设绿色生态文明村为载体，大力推进村镇规划和村庄绿化整治，建设宜居城市、宜居城镇、宜居村庄。年内创建省卫生村30条，建设生态文明村600条，农村沼气池3000座。

四、加快农村改革发展，提升农民持续增收能力

促进农村改革。按照“争当全省农村改革发展试验区”的要求，认真落实中央和省关于农村改革的各项措施，稳定和完善农村基本经营制度，积极推进农村土地承包经营权流转。做好农村土地确权、登记、发证工作，防范化解农村土地纠纷。健全农村宅基地管理制度，完善多元化的征地补偿办法，推动建立城乡统一的土地市场。稳步开展农村集体林权制度改革。积极探索城乡统筹综合配套改革，争取列入省的试点。

促进现代农业发展。在确保完成粮食播种面积和粮食产量任务的基础上，推进以温氏为龙头的全国畜牧业产业化示范中心创建工程，扶持现有农业产业化经营组织发展壮大，培育一批新的种养型、加工流通型农业龙头企业和农民专业合作组织，促进农业产业化发展。加强与珠三角发达地区合作，加快现代农业园区和优势农产品基地建设；瞄准港澳及珠三角大中城市的需求，大力发展高效绿色农业，促进农业结构调整。加强现代标准农田建设、病险水库改造和补充耕地开发等工作，改善农业生产条件。加强重大动植物疫病防控和农产品质量安全监管, 健全农业社会化服务体系，推进农业生产标准化。

促进农民持续增收。加大对“三农”投入，不折不扣地落实中央提高粮食最低收购价格、农资综合和粮食直补、良种补贴、农机具购置补贴等惠农政策。积极拓展农产品流通渠道，抓好农产品流通市场建设，稳定农产品价格。挖掘农业内部增收潜力，继续抓好智力扶贫和农村劳动力培训转移工程，引导农民参与农业产业化经营，开展生态、休闲、观光等多种形式的农业经营活动；推进农村富余劳动力转移就业，支持农民工特别是返乡农民工自主创业和灵活就业，多形式、多渠道增加农民收入。

五、加快第三产业发展，提升经济发展活力

发展商贸物流业。加快城乡商业网络规划建设，继续推进“万村千乡”市场工程，全面启动家电下乡活动，拉动城乡消费。重点发展一批连锁超市和中高级批发市场，在城区规划建设大型购物广场，引进国内外知名连锁商贸企业进驻。充分利用我市不断完善的交通条件，在沿江码头、铁路车站、高速公路出口规划建设现代物流园区，引进和培育大型专业物流企业。

发展特色旅游业。加大旅游资源整合和宣传推介力度，加强与省内外知名旅游机构合作，发展六祖朝圣、观光渡假、温泉休闲、乡村民俗等特色旅游，年内完成禅宗文化产业园整体规划。加大景区评A、酒店评星工作力度，提升旅游服务层次，力争年内新增一批国家4A级旅游景区（点）和四星级标准以上酒店，发展一批颇具特色的乡村旅游、生态旅游、工业旅游和农业旅游示范点，打造粤西旅游休闲基地。

发展房地产等其他服务业。积极扶持房地产业健康发展，加快臻汇园等12个主要房地产项目建设，年内启动城西新区规划、征地等工作。推进电信网、广播电视网、互联网“三网”融合，实现信息服务双向互动，年内完成“金保工程”、党政内网和电子监察系统二期工程建设。加快文化传媒、社区服务、中介服务、公共服务、科教服务等行业的发展，为促进生产发展、改善群众生活提供更好的服务。

六、加强统筹发展与制度创新，促进经济科学发展

强化统筹发展理念。深入开展学习实践科学发展观活动，形成继续解放思想、推动科学发展的共同意志和强大动力。把统筹全市资源环境城乡区域发展作为我市落实科学发展观的根本方法，坚持以人为本、生态优先、总揽全局、突出重点，科学谋划全市产业发展布局与体系，积极探索并大胆尝试科学发展新模式，切实转变经济增长方式，促进资源与环境、城市与农村、经济与社会、人与自然协调发展，决不走高投入、高消耗、高污染和低效益的老路。

强化规划引领作用。结合《珠江三角洲地区改革发展规划纲要》，抓紧制定并全面实施《云浮市改革发展规划纲要》，以主体功能区规划为基础,协调好国民经济和社会发展规划、土地利用总体规划、城乡规划的关系，争取列入省的“三规合一”试点；继续抓好编制和完善产业发展、交通建设及物流发展等其它专项规划，抓紧转化规划成果，促进城乡协调发展、共同繁荣。

强化制度改革创新。加快建立“三规合一”机制，促进与之相配套的行政管理体制改革。加快财税体制改革，建立县镇统一的公共财政体系，促进公共财政城乡均等化。统筹规划市内交通、园区、项目建设和教育、医疗等公共基础设施建设，促进财税收益与公共服务共建共享。加快建立健全公众参与机制、公务员考核评价机制及相关配套制度，以改革创新促进科学发展。

七、加强招商引资和外经贸工作，拉动经济持续增长

加大招商引资工作力度。充分利用现代信息手段及时宣传我市招商引资、承接产业转移的优惠政策、优势产业和优质项目。加强与珠三角、港澳台地区及国内外企业、行业商会的沟通联系，围绕我市产业规划和园区建设，大力推动统一招商、合作招商，发展代理招商、以商引商、以政引商和以侨引商。积极参加国家和省组织的各类经贸活动，并在香港、佛山及广州、深圳、东莞、中山、珠海等地区分别举办专场招商推介活动，办好第六届国际石材科技展览会。完善招商引资工作责任制和激励机制，加强对招商引资项目的跟踪服务，在谈项目促签约、签约项目促动工、动工项目促投产，不断增强经济发展后劲。

加大对外贸易工作力度。积极应对国际金融危机对企业的影响，用足用活国家支持出口政策，为出口企业提供更多的金融、信息、法律和中介等服务，鼓励和支持民营企业获取出口经营权，扩大出口。推进加工贸易企业扩大国内采购和产品内销，支持出口重点行业打造区域品牌，引导骨干企业打造自主品牌。巩固港澳台、东南亚、欧美日等传统市场，大力开拓非洲、南美、中东和俄罗斯等新兴市场。继续推进“大通关”建设，加快完善口岸配套设施，优化口岸通关环境，年内动工建设云浮出入境检验检疫中心。鼓励企业扩大先进技术设备进口，积极引进先进适用技术和管理经验。

八、加强财税金融工作，保障经济稳健运行

增强财政保障支撑能力。把培植财源、增加税收和增收节支放在财税工作的突出位置，切实提高财政保障能力。加强税源监控与征收，抓好国有资产、城市公共资源经营收益管理工作，提高财政收入总量和质量。完善公共财政制度，落实积极的财政政策，加大财政对重大项目、新农村建设和社会事业、民生工程等的投入。继续完善部门预算、政府采购、国库集中支付、财政支出绩效评价制度，深化预算执行审计、效益审计和经济责任审计、专项资金审计，提高财政资金使用效益。

增强金融服务发展能力。加强与各金融机构的战略合作，重点抓好城市、园区、交通等基础设施项目融资。培育和壮大中小企业信用贷款担保机构，继续推动企业改制上市，重点扶持温氏集团、凌丰集团、惠沄化工等企业上市融资，千方百计帮助中小企业解决融资难问题。加快农信社改革发展，做好城市信用社退出市场收尾工作。完善农村金融服务体系，积极推进小额贷款公司、村镇银行和政策性农业保险试点。支持保险机构探索建立新险种，做大保险业务。

九、加强和谐社会建设，推动经济与社会互动协调发展

抓好劳动就业和社会保障工作。进一步做好促进就业工作，年内力争新增就业岗位3万个，转移农村劳动力5万人。加强社会保险扩面征缴，完善养老保险市级统筹；建立被征地农民社会保障制度，探索农村居民基本养老保险制度；适当提高低保标准和五保供养标准，今年逐步将农村家庭年人均纯收入低于1500元的困难群众纳入低保范围，社会孤儿全部按当地五保供养标准给予保障。大力发展福利、慈善和残疾人事业。

抓好科技、教育事业。加强企业自主创新，鼓励企业加大研发投入；积极引进和推广科研成果，提升与高校及科研院所合作层次，推进佛山（云浮）产业转移工业园产学研与科技成果转化基地及省级工程技术中心建设；加强科普宣传，提高全民科技素质。进一步落实“以县为主”的义务教育管理体制，巩固普及九年义务教育成果，开展新一轮义务教育学校布局调整；加快普及高中阶段教育步伐，大力发展中等职业教育，确保毛入学率达到65%以上；加强师资队伍建设，解决代课教师问题，落实教师待遇；争取市特殊学校秋季招生开学。

抓好文化、卫生、体育等事业。加强公民思想道德建设，深入推进群众性精神文明创建活动。抓好文化信息资源共享、农家书屋、文化下乡等文化惠民工程和乡镇综合文化站建设；擦亮禅宗六祖文化、南江文化、云石文化品牌；做好第三次全国文物普查的田野调查；推进文化事业体制改革，加强文化市场管理，加快发展广播电视事业和文化产业；办好庆祝建国60周年和建市15周年系列文化活动。稳妥推进医药卫生体制改革工作；巩固和发展新型农村合作医疗和城镇居民基本医疗保险成果，积极推进全市城乡居民医疗保障全覆盖；推进国家重点中医院和全国农村中医工作先进市创建工作，完善农村三级医疗卫生服务网，年内完成20间薄弱卫生院改造和200间农村卫生站规范化建设；加强城市社区卫生服务体系建设，提升市直医疗机构综合服务能力。完善城乡体育设施建设，抓好省第十三次运动会备战参战工作。创新人口和计划生育服务管理机制，争创国家和省优质服务先进单位；继续抓好出生人口性别比偏高问题综合治理，稳定低生育水平，提高人口素质。

抓好其它各项民生工作。年内建设廉租住房260套，缓解城镇低收入家庭住房困难。争取解决农村14.45万群众饮水安全问题，全面完成省人大议案安排的81条水库移民村改造建设和190宗农村机电排灌工程建设任务。改善农村客运条件，争取农村客运通达率达90%以上。加强污染减排工作，推广资源综合利用和清洁生产，确保完成年度总量减排任务。加强城乡防灾减灾工作。大力整顿和规范市场经济秩序，严格食品药品市场准入和监管。落实安全生产责任制，坚决遏制重特大安全事故发生。扎实推进平安云浮建设，严厉打击各类违法犯罪活动，加大打黑除恶专项工作力度，坚决查处“黄赌毒”；完善社会治安防控体系，集中整治突出治安问题和治安混乱地区；强化应急处突力量建设。巩固和发展“双拥”工作成果，高标准抓好基层人武部“四个基本”建设，不断推动国防后备力量建设。深化基层群众民主自治，继续推进城乡“六好”平安和谐社区创建工作。开展社区矫正试点。加强法律援助、司法救助、人民调解和行政调解工作。落实信访工作责任制和领导包案制度，切实解决群众信访反映的各种问题。

十、加强政府自身建设，优化经济发展环境

提高科学决策、依法行政水平。根据上级部署，积极推进市县政府机构改革和事业单位分类改革，进一步理顺政府、社会和市场及政府部门之间的关系，强化政府创造良好社会环境、提供优质公共服务、维护公平正义的职能。健全行政决策程序，创新行政执法机制，完善符合科学发展要求的政府绩效评价指标体系和评估机制。主动向市人大及其常委会报告工作和向市政协通报情况，认真办理人大议案、建议和政协提案。主动听取各民主党派、各人民团体和无党派人士意见。加强法制宣传教育，认真执行《行政许可法》等法律法规，落实行政首长问责制和行政过错追究制。继续通过行风评议、政风行风热线、市长热线、城市论坛等形式，扩大公众参与渠道，推动政府工作科学化、民主化、法治化、规范化。

提高服务企业、服务基层水平。牢固树立服务理念，认真贯彻《政府信息公开条例》，加强政府管理信息共享，开展第四轮行政审批制度改革，减少办事环节，明确办事时限，提高办事效率。创新服务方式，进一步完善电子政务、电子监察、网上审批和“一条龙、一站式”服务。改进工作作风，加强规划引导、制定政策、统筹协调，深入基层、深入企业，帮助企业和群众解决生产经营和实际工作中遇到的突出困难。

提高勤政廉政、干净干事水平。加强工作督查与考核，坚决制止不作为、乱作为和办事拖沓、效率低下、铺张浪费、“吃拿卡要”等现象；坚决纠正观望等待、敷衍塞责、有令不行、有禁不止，上有政策、下有对策等行为。加强对工程项目和资金管理情况的监督检查，坚持勤俭办事，反对铺张浪费。全面落实“一岗双责”反腐倡廉责任制，建立健全惩治和预防腐败体系。加强政府效能建设，努力建设一支开拓进取、团结务实、奋发创业、勤政廉政的公务员队伍。

此外，继续加强物价、统计、人事人才、编制、经济发展研究、知识产权、民族宗教、档案、外事侨务、台务、港澳事务、人民防空、地方志、消防、保密、气象、扶贫、工会、共青团、妇联、侨联、文联、残联、社科联、工商联、红十字会、贸促会以及驻市外机构等工作。

各位代表！新形势、新目标催人奋进。站在新的历史起点上，展望云浮科学发展的美好未来，我们满怀信心。让我们更加紧密地团结在以胡锦涛同志为总书记的党中央周围，在省委、省政府和市委的正确领导下，全面贯彻落实科学发展观，解放思想、开拓创新、抢抓机遇、加快发展，全力推进广东富庶文明大西关建设进程，以更加辉煌的成就迎接建国60周年和建市15周年！
